# Potential antitumor effects of nitrogen-containing bisphosphonate in hormone receptor negative breast cancer patients with bone metastases

**DOI:** 10.1186/1471-2407-9-154

**Published:** 2009-05-20

**Authors:** In Hae Park, Jungsil Ro, Byung Ho Nam, Youngmi Kwon, Keun Seok Lee

**Affiliations:** 1 Center for breast cancer, National cancer center, Goyang-si, Korea; 2 Center for clinical trial, National cancer center, Goyang-si, Korea

## Abstract

**Background:**

This retrospective study evaluated, according to hormone receptor status, the antitumor effects of bisphosphonate especially on survival and disease progression in breast cancer patients with metastatic bone disease.

**Methods:**

Of 317 patients with initial bone metastasis and known breast cancer subtypes, 230 patients (72.6%) had hormone receptor (HR) positive tumors, and 87 patients (27.4%) had HR negative tumors. We assessed the primary outcome of overall survival (OS), after adjusting for other factors, comparing a group that received bisphosphonates (BPs) with a group that did not receive it.

**Results:**

87.8% of HR positive and 69.0% of HR negative patients received BPs with a median number of 17.7 cycles. Although BPs treatment made no survival benefit in HR positive group, HR negative patients showed a significant prolonged survival when they received BPs treatment (hazard ratio = 0.56 [95% CI 0.34 to 0.91], *P *= 0.019). In multivariate analysis, disease free interval > 2 years (*P *= 0.036), a sum of metastatic sites < 3 (*P *= 0.034), and BP treatments (*P *= 0.007) were significant factors for survival in HR negative patients.

**Conclusion:**

Bisphosphonate treatment can result in a survival benefit in metastatic breast cancer patients with HR negative tumors.

## Background

Bone metastases are common in patients with advanced breast cancer. The median survival time after the diagnosis of bone metastases is approximately two years, although it may increase with new treatment regimens [1]. As patients survive longer, the incidence of associated problems with metastatic bone disease (MBD), such as pathologic fracture or spinal cord compression, increases, and these complications may substantially reduce a patient's quality of life [1,2]. Over the past decade, bisphosphonates (BPs) have become the standard therapy for breast cancer patients with bone metastasis. It is now clear that the bisphosphonates reduce both the symptoms and complications of bone involvement [3]. In addition, the anti-tumor activities of BPs have been explored, particularly with zoledronate and pamidronate, through *in-vitro *as well as *in-vivo *animal studies [4-6]. These preclinical studies showed that BPs could reduce visceral metastasis as well as bone metastasis. However, the clinical relevance of the antitumor effects of BPs has not been firmly established. A few small pilot studies investigating the potential roles of BPs in the adjuvant setting were conducted, and their results regarding the antitumor effects of BPs were controversial, due to the non-randomized nature of these studies [7-9].

Recently, Gnant et al. reported that zoledronate, in an adjuvant setting, significantly prolonged disease-free survival beyond the time achieved with endocrine therapy alone (hazard ratio = 0.64, *P *= 0.01) [10]. Now, several large randomized controlled trials, such as the B-34 trial, the Adjuvant Zoledronic Acid to Reduce Recurrence (AZURE) trial, and the SWOG 0307 trial, are being conducted to clarify the roles of BPs in the adjuvant setting. These studies will assess the direct or indirect antitumor effects of BPs, according to the rate of bone metastasis, other visceral metastasis, and overall survival.

The antitumor effects of BPs have not been investigated in the metastatic setting, although BPs are now widely used in metastatic breast cancer. Most studies focused on the development of skeletal related events (SREs) and the quality of life of patients with metastatic bone disease [11]. In this retrospective study, we address the antitumor effect of BPs in breast cancer patients with metastatic bone disease. Our primary objective is to assess the association between BP treatment and overall survival (OS), while adjusting for other factors. The secondary objective is to evaluate the effects of BPs in preventing SREs and the progression of metastatic bone disease.

## Methods

### Study population and study assessments

This retrospective study included a total of 317 breast cancer patients with initial bone metastases, whose hormone receptor (HR) status and HER2 status were known, and who were treated between June 2001 and July 2007 at the National Cancer Center Hospital, Korea. Of these, 87 patients (27.4%) had HR negative tumors. A tumor was considered to be HER2 positive if the primary or metastatic tumor was scored 3+ by HER2 immunohistochemistry (IHC) or by amplification of the HER2 gene by the method of fluorescent in situ hybridization (FISH). If a tumor's score was 2+ by IHC, the tumor was reanalyzed using FISH. Metastatic bone disease (MBD) was diagnosed mostly by means of radionuclide bone scans performed for follow-up. Magnetic resonance imaging was done when patients showed skeletal related symptoms such as pain, fractures, or neurologic signs with equivocal bone scan results.

Patients with bone metastases were followed up until August 2008, and their medical records were reviewed for clinical data, including age at the initial diagnosis, age at diagnosis of disease recurrence, initial stage of the disease, pathological type, disease-free interval, number and site of metastases, treatment after diagnosis, especially bisphosphonate treatment, and survival interval from the diagnosis of disease recurrence or initial stage IV presentation.

The administered bisphosphonates (BPs) differed according to the periods of treatment, compliance, tolerability, and insurance strategy. The types of bisphosphonate used were zoledronate or pamidronate. These drugs were given at three or four week intervals. Patients who received more than three consecutive months of bisphosphonate treatment were designated as members of the bisphosphonate group (BP group), and other patients were designated as members of the non-bisphosphonate group (non-BP group).

For the evaluation of the bisphosphonate effect on bone metastasis, we assessed the progression of bone metastasis, time to progression of bone disease (TTP_BD), and development of skeletal related events (SREs). SREs were defined as pathological fractures, or surgery or palliative radiation to bone in order to treat or prevent impending fractures or cord compressions. Disease progression in bone was defined as the appearance of any new bone lesions, or the progression of existing bone metastases, including the development of SREs. To monitor the side effects of bisphosphonate, the serum levels of creatinine and total calcium were regularly checked. Bisphosphonate treatment was temporarily stopped before and after dental procedures. Patients undergoing bisphosphonate treatment were instructed to take an oral calcium supplement containing vitamin D.

This study protocol was approved by the Institutional Review Board for the National Cancer Center (IRB protocol number NCCNCS 08-195). Because this was a retrospective analysis that involved no additional risk to patients, the Institutional Review Board approved a waiver of informed consent.

### Statistical methods

Descriptive statistics, including sample size, median, and range, were reported for continuous variables. Discrete variables were summarized using frequencies and percentages. Clinical parameters and treatment response were compared using 2-way tables, chi-square, and Mann-Whitney U test. Time to progression of bone disease (TTP_BD) and overall survival (OS) were estimated by Kaplan-Meier analysis and compared using the log-rank test. Cox proportional hazard model was used to identify the independent predictive factors that significantly influenced the overall survival of patients with metastatic bone disease. The proportionality assumption for the Cox regression and the log rank test was checked and verified by using the log-log plot. All *P *values were 2-tailed, with 5% significance levels. All statistics were calculated using SPSS^® ^13.0 software (SPSS Inc., Chicago, IL, USA).

## Results

### Baseline characteristics

Among 317 patients with bone metastases at diagnosis, 262 (82.6%) patients were treated with BPs during follow-up. The median number of cycles of BP treatment was 17.7 (range, 3 to 57 cycles), and the median interval between cycles was 31 days. Patients' characteristics are shown in Table 1. Types of BP were zoledronate (27.5%), pamidronate (40.1%), and ibandronate (0.8%). Eighty-three (31.6%) patients received more than one type of BP, sequentially. Aside from more weight bearing bone involvement (*P *< 0.001), the BP group had better prognostic factors, such as HR positivity (*P *< 0.001), negative HER2 receptors (*P *= 0.035), fewer visceral metastases (*P *= 0.002), bone only metastases (*P *= 0.014), and fewer metastatic sites (*P *= 0.006), compared to the non-BP group. There was no significant difference in use of anti-HER2 therapy between BP and non-BP groups.

**Table 1:** Characteristics of total patients with metastatic bone disease

Factors	BP group N = 262	Non-BP group N = 55	***P*-value**^||^
Age, median years (range)	45.5 (25–77)	50 (30–68)	NS*
PS** (ECOG ≥ 2)	10/166 (6.0%)	2/27 (7.4%)	NS
Pre-menopausal	79/176 (44.9%)	7/30 (23.3%)	0.027
Post-menopausal	97/176 (55.1%)	23/30 (76.7%)	
ER***/PR^† ^positive	202 (77.1%)	28 (50.9%)	< 0.001
HER2 positive	72 (27.5%)	23 (41.8%)	0.035
DFI^‡ ^(month, median, range)	25 (0–230)	21 (0–174)	NS
Initial stage IV	61 (23.4%)	11 (20%)	NS
Adjuvant treatment			
Radiotherapy	111 (42.4%)	27 (49.1%)	NS
Chemotherapy	176 (67.2%)	38 (69.1%)	NS
Hormonal therapy	127 (48.5%)	21 (38.2%)	NS
Metastatic sites			
Visceral (liver, lung)	116 (44.3%)	37 (67.3%)	0.002
LNs^§^	119 (45.4%)	30 (54.5%)	NS
Soft tissue	78 (29.8%)	20 36.4%)	NS
Bone only	81 (30.9%)	8 (14.5%)	0.014
Weight bearing bone involvement	225 (86.2%)	25 (45.5%)	< 0.001
Sum of metastatic sites (range)	2 (1–6)	3 (1–6)	0.006
Cycles of palliative chemotherapy			NS
1	36 (13.7%)	8 (14.5%)	
2	53 (20.2%)	13 (23.6%)	
≥ 3	157 (59.9%)	29 (52.7%)	
Palliative AI^¶ ^use	168 (64.1%)	23 (41.8%)	0.002
Palliative anti-HER2 therapy	58/72 (80.6%)	20/23 (86.9%)	NS
Disease progression in bone (n)	97 (47.8%)	21 (38.2%)	NS
SREs (except the first event)	63 (27.2%)	21 (24.7%)	NS

Abbreviations:*, not significant; **, performance status; ***, estrogen receptor; †, progesteron receptor;

‡, disease free interval; § lymph nodes; ¶ aromatase inhibitor; ||, *P*-value was obtained by chi-square test except variables of age and DFI which were obtained through Mann-Whitney U test

**Figure 1:**
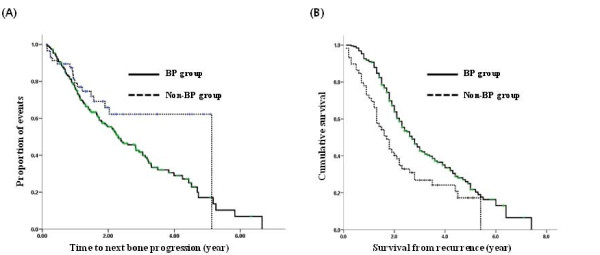
**Total patients with metastatic bone disease**. (A) TTP_BD (*P *= 0.059), (B) OS (P < 0.001).

The log-log plot showed that the proportionality assumption was satisfied (data not shown). Although the rate of progression of bone disease and the incidence of SREs (except for the first event) were higher in the BP group, there was no statistical difference in the time to progression of the bone disease (TTP_BD) according to BP treatment (*P = *0.059). The overall survival (OS) was significantly longer in the BP group (Fig. 1).

In the univariate analysis, the following factors, in addition to BP treatment, showed positive association with longer survival: HR positivity, HER2 negativity, disease free interval (DFI) > 2 years, sum of metastatic sites < 3, and no visceral involvement (Table 2). Among these factors, HR positivity, DFI > 2 years, and no visceral metastasis were significant in multivariate Cox analysis.

**Table 2:** Analysis for OS of total patients

	Univariate		Multivariate	
	
Variables	Hazard ratio (95% CI)	*P*-value	Hazard ratio (95% CI)	*P*-value
Age <40	0.86 (0.66–1.13)	0.29	-	-
Post-menopausal state	1.11 (0.77–1.59)	0.59	-	-
DFI* >2 yr	0.63 (0.48–0.83)	0.001	0.65 (0.48–0.86)	0.003
Initial stage IV	0.93 (0.67–1.29)	0.66	-	-
ER**/PR*** positive	0.36 (0.27–0.48)	< 0.001	0.41 (0.31–0.56)	< 0.001
HER2 positive	1.50 (1.13–1.98)	0.005	0.94 (0.69–1.28)	0.69
Visceral metastasis	2.02 (1.55–2.65)	< 0.001	1.93 (1.39–2.68)	< 0.001
Sum of metastasis sites ≥ 3	1.53 (1.17–2.00)	0.002	0.99 (0.73–1.37)	0.96
BP^† ^use	0.52 (0.37–0.73)	< 0.001	0.70 (0.50–0.98)	0.96

Abbreviations: *, disease free interval; **, estrogen receptor; ***, progesteron receptor; †, bisphosphonate;.

It has been known that patients with hormone receptor (HR) positive tumors tend to develop more bone metastasis than those with HR negative tumors. As shown in Table 1, our study population reflected this trend. When we conducted the survival analysis of all patients, longer survival was associated with BP treatment as well as with HR positivity (Table 2). We therefore analyzed the effect of BP treatment on survival after stratifying HR status into HR positive and HR negative groups because we wanted to exclude the possible role of HR positivity as a confounding factor.

### The effect of BP treatment in the HR positive group

**Figure 2:**
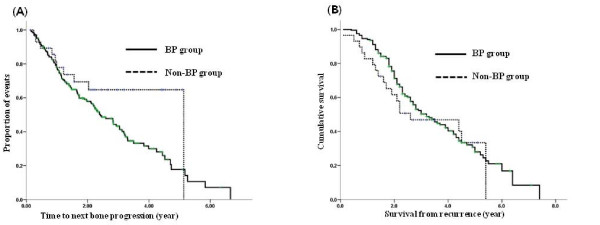
**Patients with HR positive metastatic bone disease**. (A) TTP_BD (*P *= 0.117), (B) OS (*P *= 0.104).

Of the 230 patients whose tumors were HR positive, 202 patients (87.8%) received BP treatments. Although more patients in the BP group developed disease progression in bone and of SREs (except for the first event), these associations were not statistically significant (Table 3). There was no significant difference in TTP_BD or OS associated with BP treatment (*P *= 0.117, *P *= 0.104 respectively) (Fig. 2).

**Table 3:** Characteristics of patients with HR positive and HR negative tumor

	HR* positive tumor			HR negative tumor		
	
Factors	BP group N = 202	Non-BP group N = 28	***P*-value**^||^	BP group N = 60	Non-BP group N = 27	***P*-value**^||^
Age (yr, median, range)	46 (28–77)	50.5 (33–68)	NS**	43.5 (25–69)	50 (30–67)	NS
PS*** (ECOG ≥ 2)	9/138 (6.5%)	0/16 (0%)	NS	1/28 (3.6%)	2/11 (18.2%)	NS
Pre-menopausal	66/144(45.8%)	4/18 (22.2%)	0.057	13/32 (40.6%)	3/12 (25%)	NS
Post-menopausal	78/144(54.2%)	14/18 (77.8%)		19/32 (59.4%)	9/12 (75%)	
HER2 positive	44 (21.8%)	12 (42.9%)	0.015	28 (46.7%)	11 (40.7%)	NS
DFI^† ^(months, median, range)	27.5 (0–230)	26 (82.1%)	NS	18.5 (0–86)	18 (0–174)	NS
Initial stage IV	44 (21.8%)	4 (14.3%)	NS	17 (28.8%)	7 (25.9%)	NS
Adjuvant treatment						
Radiotherapy	82 (40.6%)	15 (53.6%)	NS	29 (48.3%)	12 (44.4%)	NS
Chemotherapy	139 (68.8%)	21 (75%)	NS	37 (61.7%)	17 (63%)	NS
Hormonal therapy	114 (56.4%)	17 (60.7%)	NS	13 (21.7%)	4 (14.8%)	NS
Metastatic sites						
Visceral (liver, lung)	80 (39.6%)	18 (64.3%)	0.013	36 (60%)	19 (70.4%)	NS
LNs^‡^	87 (43.1%)	12 (42.9%)	NS	32 (53.3%)	18 (66.7%)	NS
Soft tissue	57 (28.2%)	8 (28.6%)	NS	21 (35%)	12 (44.4%)	NS
Bone only	73 (36.1%)	6 (21.4%)	NS	8 (13.3%)	2 (7.4%)	NS
Weight bearing bone involvement	174 (86.6%)	13 (46.4%)	< 0.001	51 (85%)	12 (44.4%)	< 0.001
Sum of metastatic sites (range)	2 (1–6)	3 (1–5)	NS	3 (1–5)	3 (1–6)	NS
Cycles of palliative chemotherapy			NS			NS
1	27 (13.4%)	4 (14.3%)		9 (15%)	4 (14.8%)	
2	43 (21.3%)	3 (10.7%)		10 (16.7%)	10 (37%)	
≥ 3	116 (57.4%)	17 (60.7%)		41 (68.3%)	12 (44.4%)	
Palliative AI^§ ^use	159 (78.7%)	21 (75%)	NS	9 (15%)	2 (7.4%)	NS
Palliative anti-HER2 therapy	35/44 (79.5%)	10/12 (83.3%)	NS	23/28 (82.1%)	10/11 (90.9%)	NS
Disease progression in bone	96 (47.5%)	10 (35.7%)	NS	23 (38.3%)	6 (22.2%)	NS
SREs (except the first event)	59 (29.2%)	4 (14.3%)	NS	15 (25%)	4 (14.8%)	NS

Abbreviations: *, hormone receptor; **, not significant; ***, performance status; †, disease free interval; ‡, lymph nodes;

§ aromatase inhibitor; ||, *P*-value was obtained via chi-square test except variables of age and DFI which were obtained through Mann-Whitney U test.

HER2 receptor positivity and visceral organ involvement were important factors in OS. These results were confirmed through multivariate analysis. However, BP treatment was not a significant component for OS in HR positive patients (Table 4).

**Table 4:** Analysis for OS of patients according to hormone receptor status

	HR positive patients				HR negative patients			
	
	Univariate		Multivariate		Univariate		Multivariate	
	
Variables	Hazard ratio (95% CI)	*P*-value	Hazard ratio (95% CI)	*P*-value	Hazard ratio (95% CI)	*P*-value	Hazard ratio (95% CI)	*P*-value
Age < 40 yr	0.85 (0.60–1.19)	0.344	-	-	0.75 (0.47–1.21)	0.237	-	-
Postmenopausal state	1.26 0.81–1.94)	0.302	-	-	-	-	-	-
HER2 positive	1.66 (1.15–2.40)	0.007	1.54 (1.06–2.23)	0.022	0.67 (0.43–1.06)	0.088	-	-
DFI* > 2 yr	0.76 (0.54–1.07)	0.12	-	-	0.61 (0.38–0.98)	0.040	0.59 (0.36–0.97)	0.036
Initial stage IV	0.76 (0.49–1.19)	0.232	-	-	0.96 (0.58–1.59)	0.882	-	-
Sum of metastasis sites ≥ 3	1.32 (0.94–1.83)	0.105	-	-	1.83 (1.13–2.97)	0.014	1.70 (1.04–2.78)	0.034
Visceral metastasis	1.78 (1.28–2.48)	0.001	1.7 (1.22–2.38)	0.002	-	-	-	-
BP** use	0.67 (0.42–1.19)	0.111	-	-	0.56 (0.34–0.91)	0.019	0.50 (0.30–0.83)	0.007

Abbreviations: *, disease free interval; **, bisphosphonate.

### The effect of BP treatment in the HR negative group

**Figure 3:**
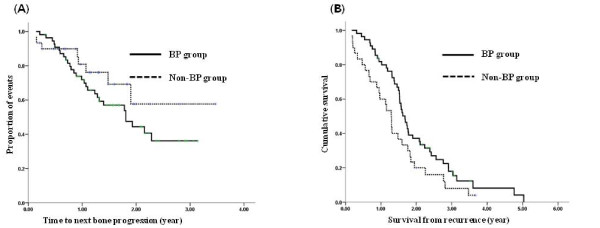
**Patients with HR negative metastatic bone disease**. (A) TTP_BD (*P *= 0.163), (B) OS (*P *= 0.040).

Of the remaining 87 patients with bone metastasis whose tumors were HR negative, 60 (69%) patients were treated with BP for metastatic bone disease. There was no significant difference in the baseline characteristics between the BP and the non-BP groups, except that more patients had weight bearing bone metastases in the BP group compared to the non-BP group (*P *< 0.001) (Table 3). The rate of disease progression in bone metastases and SREs (except for the first event) was greater in the BP group. However, the difference was not significant (*P *= 0.14, *P *= 0.29, respectively). TTP_BD was not significantly different between the two groups (Fig. 3).

Unlike the OS observed in the HR positive patients, OS was significantly prolonged in the BP group (median, 1.7 yr vs. 1.3 yr, *P *= 0.040, Fig. 3). In univariate analysis for OS, disease free interval > 2 years (hazard ratio = 0.61 [95% CI 0.38 to 0.98], *P *= 0.040), a sum of metastatic sites ≥ 3 (hazard ratio = 1.83 [95% CI 1.13 to 2.97], *P *= 0.014), and BP treatments (hazard ratio = 0.56 [95% CI 0.34 to 0.91], *P *= 0.019) were significant factors for survival in HR negative patients. These results were verified through multivariate analysis (Table 4).

## Discussion

BPs have been used for more than 15 years to improve the outcomes of patients with bone metastases from solid tumors, as well as hematologic malignancies. In bone metastases, the cancer cells invade the bone marrow cavity and produce parathyroid hormone-related protein (PTH-rP), which stimulates osteoclastic resorption by increasing the production of the receptor activator of the nuclear factor-κB ligand (RANKL) by osteoblast and stromal cells. The RANKL binds to its receptor, RANK, on osteoclast lineage cells, induces differentiation into mature osteoclasts, and stimulates osteoclast activity [12,13]. Nitrogen-containing BPs (N-BPs) such as zoledronate, pamidronate, and ibandronate affect osteoclast activity and survival through inhibiting farnesyl diphosphonate synthase in the mevalonate pathway [13]. This inhibition also seems to account for the antitumor effects of N-BPs *in vitro *[14]. N-BPs also have anti-angiogenic effects, decreasing the serum VEGF of patients with a variety of solid tumors [15]. Inhibition by N-BPs enhanced the antitumor activity of known cytotoxic agents that were commonly used in clinical settings [16]. Among various BPs, N-BPs, especially zoledronate, have the strongest antitumor effect *in vitro *[17].

In contrast to the consistent preclinical antitumor effects of BPs, the results of clinical studies in breast cancer have shown conflicting findings. Diel and Powles indicated that clodronate treatment prevented skeletal metastases with no effect on visceral metastases, and improved overall survival [7,18-20]. However, Saarto reported that clodronate treatment worsened survival, especially in hormone negative patients, among whom significantly more patients on clodronate experienced non-skeletal metastases than among the control group [8].

In this study, we investigated the effect of BP in a metastatic setting, specifically in patients with metastatic bone disease from breast cancer. The most important limitation of this retrospective study is that the use of BPs was based on the clinical decision to prevent complications such as pathological fracture, bone pain, and hypercalcemia. Patients who had experienced SREs secondary to bone metastasis tended to be put on bisphosphonate more often. Consequently, patients with BP group in this study developed more often progression of bone disease and SREs, because BP treated group had more advanced and complicated bone disease at the outset of BP use. Conversely, patients with bone metastasis who did not have symptoms or had only minimal tumor burden tended not to receive BP treatment. Therefore, patients with more indolent bone disease had greater propensity to be included in the non-BP group. With these inherent unbalances, TTP_BD was not significantly different according to BP treatment in both HR positive and HR negative groups.

In the first analysis, including all patients with metastatic bone disease, favorable OS was expected in the BP group, as a result of an imbalance in hormone receptor status, the presence of visceral organs involvement, and other important prognostic factors. It was still possible that BP treatment was an important factor for survival because far more patients with HR positive tumors received BP treatment. In the next analysis, we corrected for this confounding factor by looking separately at patients with HR positive tumors within the BP-treated vs. untreated groups. In these patients, HER2 positivity and visceral organ involvement were important factors for OS, but there was no significant difference in OS with or without BP treatment. In HR negative patients, on the contrary, an OS difference was significant in favor of the BP treatment. HR negative patients' characteristics were well balanced between the BP treated and untreated groups. There was no concern for a possible interaction between hormonal therapy and BPs in HR negative patients.

These results are contrary to those of Saarto's clodronate study. In that study, 10-year disease free survival was much worse in the clodronate treatment group, especially in estrogen receptor (ER) negative patients [8]. It was assumed that antiestrogen might have opposed the detrimental effect of BPs. However, this proposition lacks a biological rationale. To the best of our knowledge, no published studies explored the relationship between hormone receptor status and the effect of BP treatment in patients with bone metastasis. Cross et al. observed a more frequent expression of RANKL in estrogen negative patients with a high histologic grade breast cancer. They observed a significant negative relation between the expression of RANKL and tumor necrosis factor-related apoptosis-inducing ligand (TRAIL) [21]. In other words, estrogen negative tumors showed higher RANKL expression and lower TRAIL expression. Therefore, BP treatment could be more beneficial to ER negative tumors through suppressing RANKL production by osteoblast and other stromal cells.

In this study, it may appear that there were unexpected interactions of anti-hormonal treatment with BPs in HR positive patients. Recently, studies of the role of zoledronate in preventing treatment-induced bone loss in pre- and post-menopausal breast cancer patients were performed. They showed that hormonal treatment combined with zoledronate did not influence the survival or disease progression in hormone responsive breast cancer [22,23]. Further studies are needed to fully elucidate the effects of BPs in HR positive tumors.

Another limitation of this study was that patients receiving different N-BPs were included. Variation in the N-BPs made it difficult to compare the efficiency among the N-BPs zoledronate and pamidronate. There is preclinical data showing that zoledronate is much more potent than other BPs. However, this has not been confirmed in clinical settings. Clinically, the choice among the various N-BPs is dependent on the adherence of patients and preference of clinicians [11].

## Conclusion

We conclude that BP treatment may give a survival benefit in metastatic breast cancer patients, particularly in patients with HR negative tumors, which are known to have a poorer prognosis. We believe that this analysis adds insight into the roles of BPs in metastatic breast cancer, in addition to their ancillary role in supportive care. Based on these results, new strategies could be investigated for the possible benefits of BP treatment in metastatic breast cancer patients without bone involvement.

## Abbreviations

HR: hormone receptor; BPs: bisphosphonates; N-BPs: nitrogen containing bisphosphonates; OS: overall survival; DFI: disease free interval; MBD: metastatic bone disease; SREs: skeletal related events; TTP_BD: time to progression of bone disease; IHC: immunohistochemistry; FISH: fluorescent in situ hybridization; RANKL: receptor activator of the nuclear factor-κB ligand; TRAIL: tumor necrosis factor-related apoptosis-inducing ligand.

## Competing interests

The authors declare that they have no competing interests.

## Authors' contributions

IHP and JR designed the study and conducted the data acquisition. IHP and BHN performed the statistical analysis. IHP, JR and BHN participated in the interpretation of the data. IHP and JR drafted and revised the manuscript. IHP, JR, BHN, YK and KSL participated in critical review of the manuscript. All authors read and approved the final manuscript.

## Pre-publication history

The pre-publication history for this paper can be accessed here:

http://www.biomedcentral.com/1471-2407/9/154/prepub
